# A Dendritic Mechanism for Decoding Traveling Waves: Principles and Applications to Motor Cortex

**DOI:** 10.1371/journal.pcbi.1003260

**Published:** 2013-10-31

**Authors:** Stewart Heitmann, Tjeerd Boonstra, Michael Breakspear

**Affiliations:** 1 School of Psychiatry, The University of New South Wales, Sydney, Australia; 2 The Black Dog Institute, Sydney, Australia; 3 Research Institute MOVE, VU University, Amsterdam, The Netherlands; 4 Queensland Institute of Medical Research, Brisbane, Australia; 5 Royal Brisbane and Women's Hospital, Brisbane, Australia; Stanford University, United States of America

## Abstract

Traveling waves of neuronal oscillations have been observed in many cortical regions, including the motor and sensory cortex. Such waves are often modulated in a task-dependent fashion although their precise functional role remains a matter of debate. Here we conjecture that the cortex can utilize the direction and wavelength of traveling waves to encode information. We present a novel neural mechanism by which such information may be decoded by the spatial arrangement of receptors within the dendritic receptor field. In particular, we show how the density distributions of excitatory and inhibitory receptors can combine to act as a spatial filter of wave patterns. The proposed dendritic mechanism ensures that the neuron selectively responds to specific wave patterns, thus constituting a neural basis of pattern decoding. We validate this proposal in the descending motor system, where we model the large receptor fields of the pyramidal tract neurons — the principle outputs of the motor cortex — decoding motor commands encoded in the direction of traveling wave patterns in motor cortex. We use an existing model of field oscillations in motor cortex to investigate how the topology of the pyramidal cell receptor field acts to tune the cells responses to specific oscillatory wave patterns, even when those patterns are highly degraded. The model replicates key findings of the descending motor system during simple motor tasks, including variable interspike intervals and weak corticospinal coherence. By additionally showing how the nature of the wave patterns can be controlled by modulating the topology of local intra-cortical connections, we hence propose a novel integrated neuronal model of encoding and decoding motor commands.

## Author Summary

Physiological studies in humans and monkeys have revealed spatially organized waves of neuronal activity that propagate across the cortex during sensory or behavioral tasks. However the functional role of such waves remains elusive. In the present study, we use numerical simulation to investigate whether wave patterns may serve as a basis for neural coding in cortex. Specifically, we propose a theoretical dendritic mechanism which permits neurons to respond selectively to the morphological properties of waves. In this proposal, the arrangement of excitatory and inhibitory receptors within the dendritic receptor field constitutes a spatial filter of the incoming wave patterns. The proposed mechanism allows the neuron to discriminate waves based on wavelength and orientation, thereby providing a basis for neural decoding. We explore this concept in the context of the descending motor system where the pyramidal tract neurons of motor cortex monosynaptically innervate motor neurons in the spinal cord. Pyramidal tract neurons have broad dendritic fields which make them ideal candidates for spatial filters of waves in motor cortex. Our model demonstrates how wave patterns in motor cortex can be transformed into a descending motor drive which replicates some fundamental oscillatory properties of human motor physiology.

## Introduction

**Figure 1:**
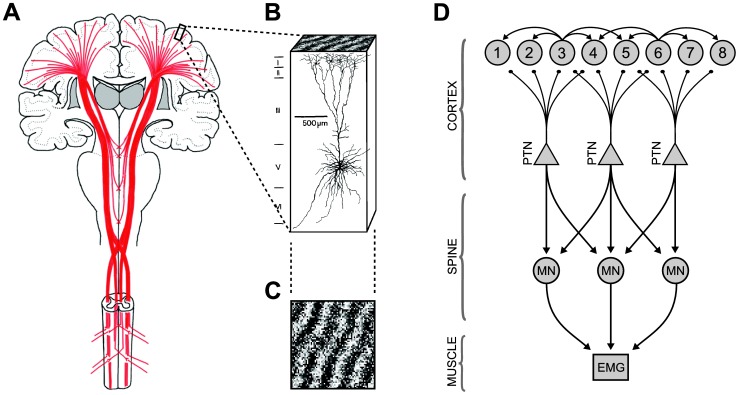
Modeling the descending motor system. (**A**) Major fiber tracts of the descending motor system, redrawn from [105]. Axons of the pyramidal tract neurons (red) descend from the motor cortex to monosynaptically innervate motor neurons in the spinal cord. (**B**) Schematic representation of the dendritic arbors of a typical pyramidal tract neuron (PTN). The apical dendrites project widely throughout the superficial layers of cortex and thus are ideally placed to detect surface wave patterns in the neural activity (top). (**C**) Simulated cortical wave pattern. (**D**) The descending motor model. Cortical wave patterns are generated by a sheet of spatially-coupled phase oscillators (circles, 1–8). These wave patterns are spatially filtered by the dendritic trees of the pyramidal tract neurons to produce an amplitude-modulated oscillatory current at the soma. Spikes initiated by the PTN are transmitted to a randomly selected pool of motor neurons (MN) in the spine. Each MN integrates the incoming spikes to produce a muscle drive spike train. Net muscle drive is quantified by simulated Electromyogram (EMG). The cortical wave model is adapted from [17]. The MN and EMG models are adapted from [41].

Traveling waves of oscillatory neuronal activity have been observed at many spatial scales although their functional role remains a matter of debate [1]. Waves have been implicated in perception [2]–[7], working memory [8], pathological seizure-like states [9], motor control [10]–[12] and neural computation [13], [14]. Waves also arise readily in neurobiological models of oscillatory activity [15], [16]. We recently proposed that the morphological properties of waves in motor cortex may serve as a neural basis for encoding movement-related information [17]. In the present study we explore how spatially-organized receptors within the dendritic field allow neurons to act as spatial filters of those wave patterns to effectively decode the information contained within their wavelength, coherence and direction. We use numerical simulation to explore this proposal in the context of the human descending motor system where we model the response of the principle output neurons of the motor cortex to simulated waves in cortex (Figure 1). The dendritic receptor field is modeled as a spatial Gabor filter which selectively initiates actions potentials in the neuron whenever it detects a specific wave pattern. Gabor filters have previously been used to characterize the receptor fields of ‘simple cells’ in visual cortex [18], [19] and here we assume that similar structures may likewise be plausible in motor cortex, giving examples of how this could be accomplished. We show that dendritic fields in cortex may serve as biological Gabor filters of internally generated patterns of oscillatory activity. Furthermore, we show how the output neurons of motor cortex may use Gabor filtering to translate those oscillatory patterns into steady motor output in the spine.

The prevailing notion of dendritic computation is credited to McCullough and Pitts [20] who were first to model dendrites as a simple linear summation of synaptic input followed by nonlinear thresholding (see [21]–[24] for reviews). Contemporary accounts have since recognized that dendritic morphology also contributes to transforming synaptic currents prior to their arrival at the cell soma [25]–[28]. Pyramidal neurons, for example, perform coincidence detection between synaptic inputs arriving on the apical and basal dendrites by exploiting transmission delays within the dendrite itself [29]. Yet dendrites are more than just tapped delay lines [30], they are active structures that are sensitive to the spatial patterning of temporal sequences along the dendritic arms [31]–[33]. The timing of spatially organized synaptic inputs is particularly likely to have implications for neural computation in oscillatory neural frameworks where the phase of a signal is paramount.

A conductance-based model of the dendrite is presented that demonstrates how spatially organized inhibitory and excitatory receptors can act in unison as a biological Gabor filter. We then present a two-compartment neural model that combines a model of the dendrite as a Gabor filter coupled with a conductance-based model of the soma. The combined model thus decodes spatial phase patterns (e.g., waves) into realistic action potentials. We apply this model to the case of pyramidal tract neurons (PTNs) which are the principle output neurons of the motor cortex. These neurons have long axons that monosynaptically innervate motor neurons and interneurons in the spine (Figure 1A). The direct corticospinal pathway is known to play a role in skilled reaching and grasping movements in higher species [34] with cell discharge rates that are primarily related to muscle force [35]. PTNs also make extensive lateral connections throughout motor cortex [36], [37] (Figure 1B) and so are ideally placed to broadly sample cortical wave activity (Figure 1C). Specifically, we consider waves in beta band (12–30 Hz) oscillations. Beta oscillations have long been implicated in the execution and planning of movement [38]–[40] but only recently has that activity also been shown to be spatially organized as waves [10]–[12]. Those waves have a spatial scale of approximately 1 cm. The scale of the proposed dendritic mechanism restricts it to wave patterns at smaller spatial scales (e.g., sub-millimeter wavelengths) than those that have thus far been reported.

The efficacy of the proposed mechanism is explored by simulating the full motor pathway from cortex to muscle (Figure 1D) using established models of motor cortex [17], motor neuron (MN) [41] and the surface electromyogram (EMG) of muscle [41]. The full model recapitulates key features of neurophysiological recordings acquired during simple purposeful motor activity, particularly the task-locked modulations in rhythmic coherence between electrocortical and electromuscular activity [42]–[45]. In doing so, we integrate two active fields of research: Traveling oscillatory waves — which encode motor commands — and dendritic computation — which leads to their decoding through spatial filters.

## Results

**Figure 2:**
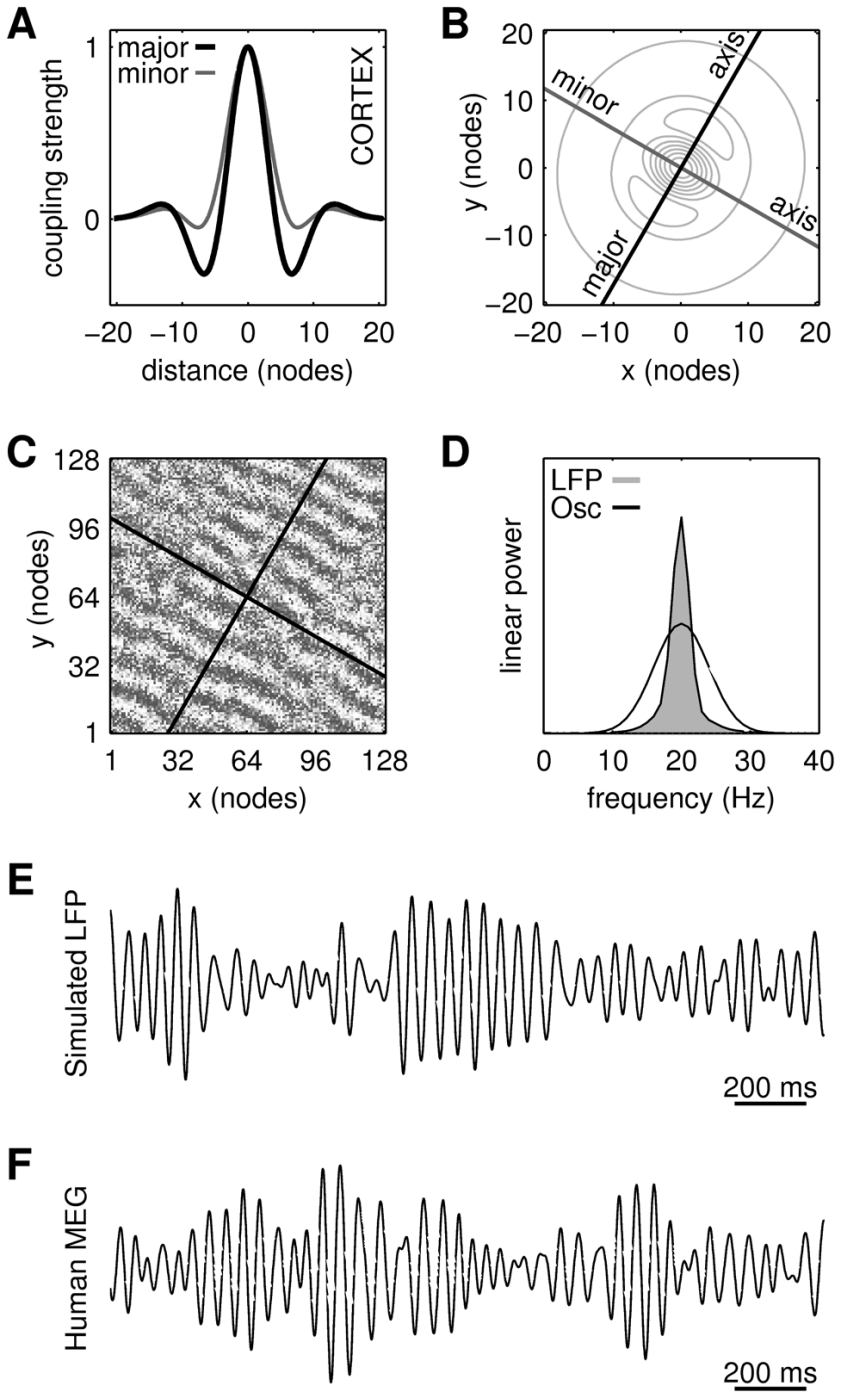
The cortical model. (**A**) Profiles of the spatial coupling kernel along its major and minor axes of orientation. (**B**) Contours of the spatial coupling kernel. (**C**) Exemplar oscillator pattern with this coupling kernel. Shading indicates phase. Black lines indicate the orientation of the kernel axes. (**D**) Frequency spectrum of the simulated local field potential (LFP) superimposed on the distribution of the autonomous oscillator frequencies (Osc). The latter is normally distributed with M = 20 Hz and SD = 4 Hz. (**E**) Time course of the simulated local field potential. (**F**) Time course of MEG signal recorded over human motor cortex during a precision grip task.

We simulated traveling waves of beta oscillations using a neurobiologically informed model of cortex [17] where coupled oscillators represent the phases of spatially distributed oscillations within a local patch of motor cortex (Figure 2). Modeling neuronal synchronization using a phase-only model is justified by the phase-reduction approximation which has a rich history in theoretical neuroscience [46]–[50]. Oscillators were spatially coupled using an anisotropic form of inhibitory-surround coupling topology to induce traveling waves of synchronization in the cortical sheet [15], [16], [51]–[53]. The resulting wave patterns were sampled by a population of randomly placed PTNs with identical receptor field morphologies. A pool of motor neurons converted the PTN output into a net muscle drive that was quantified by the simulated EMG. It is then shown that the amplitude of the final muscle drive can be controlled by varying the orientation of the cortical wave pattern with respect to the orientation of the PTN receptor fields. The results of each of these levels in our hierarchical model are now presented in sequence.

### Cortical dynamics

Cortex was modeled by a 128×128 array of spatially-coupled Kuramoto [54] oscillators (Methods, equations 2–4) where the phase of each oscillator represents the net phase of a localized patch of motor cortex [17]. This model approximates large-scale beta band oscillatory activity in cortex that is thought to be mediated by the long-range lateral connections within the superficial layers [7]. Anisotropic inhibitory-surround coupling (Figures 2A,B) has been previously reported to evoke traveling waves in this model [17] where the topology of the inhibitory surround governs the wavelength and orientation of the emergent traveling waves (Figure 2C). The resulting waves tend to propagate in either direction along the major axis of the coupling kernel. It is common for the waves to be segregated into localized patches which march coherently within each patch but in opposing directions between patches. In such cases the patches appear to be bounded by chains of spiral centers.

A broad distribution of intrinsic oscillator frequencies (M = 20 Hz, SD = 4 Hz; Figure 2D, labeled ‘Osc’) was used to achieve partial synchronization between oscillators. Partial synchronization degrades the wave pattern in a manner that resembles the effects of noise even though the governing equations are entirely deterministic [16]. This injects realistic variability into the cortical model without the need for explicit stochastic terms. That variability is most evident in the simulated LFP (Figure 2E) which exhibits an ongoing waxing and waning that does not appear to repeat periodically. Waxing and waning of oscillatory signals is routinely observed in physiology. Figure 2F shows an example of MEG oscillations in human primary motor cortex recorded during a steady hold task.

### Pyramidal Tract Neuron (PTN)

**Figure 3:**
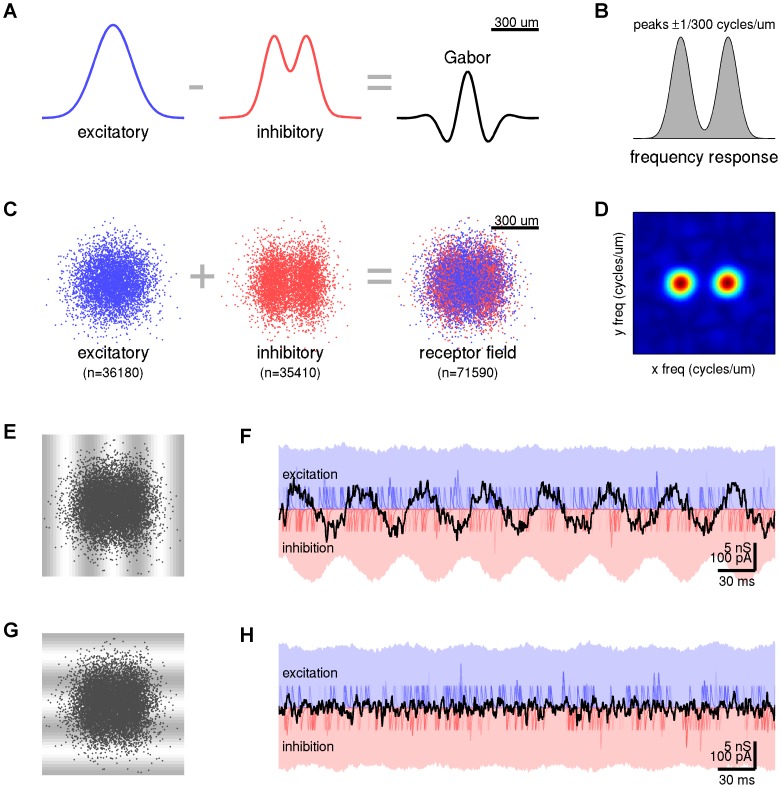
Gabor filtering by excitatory and inhibitory receptor densities. (**A**) Density profiles for excitatory (blue) and inhibitory (red) receptor populations which combine to form a Gabor filter (black). In this case, the excitatory density was nominated as Gaussian. (**B**) Spatial frequency response of the Gabor filter. Peaks correspond to waves of length 300 µm. (**C**) Excitatory (blue) and inhibitory (red) receptor samples taken from the density distributions in panel A. The combined receptor field (blue+red) represents the dendritic field of the neuron. (**D**) Spatial frequency response of the combined receptor field. Peaks correspond to vertically oriented waves of length 300 µm. (**E**) The combined receptor field superimposed on its preferred wave pattern. The wave pattern propagates from left to right at 6 mm/sec to simulate 20 Hz oscillations in the cortical field. (**F**) Time course of the net excitatory (blue shading) and inhibitory (red shading) conductances in response the preferred wave pattern. Faint lines show individual post-synaptic conductances for n = 40 randomly selected receptors (not to scale). Each receptor fires 20 spikes/sec on average. Heavy black line shows the dendritic current induced by the net changes in conductance. The amplitude of the dendritic current is modulated as the wave propagates across the receptor field. (**G**) The combined receptor field superimposed on the orthogonal wave pattern which propagates from top to bottom at 6 mm/sec. (**H**) Time course of the dendritic response to the orthogonal wave pattern. In this case the wave pattern does not modulate the dendritic current even though the individual receptors still fire at 20 spikes/sec on average.

To study the effect of the spatial arrangement of dendritic receptors on soma current, we simulated the synaptic currents flowing into the dendrite using the conductance-based model,
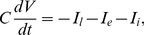
(1)where 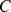 is the membrane capacitance, 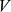 is the membrane potential, 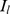 is the membrane leak current, 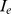 and 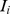 are the net synaptic currents of the excitatory and inhibitory receptor populations respectively. The spatial densities of the receptor populations were chosen so they combined to form a Gabor filter (Figure 3A). The Gabor filter was tuned to respond maximally to waves of length 300 µm (Figure 3B). Many combinations of excitatory and inhibitory densities can satisfy this requirement. Here, we nominated the excitatory density as a Gaussian distribution (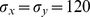 µm; peak density 0.4 synapse/µm^2^) and solved for the inhibitory distribution given a Gabor function with 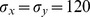 µm and a peak density of 0.2 synapse/µm^2^ (see Methods). The resulting distributions have a width of approximately 600 µm which corresponds to the width of PTN receptor fields [36], [37], [55]. The total number of synapses circumscribed by these distributions also fell within physiological estimates of 60,000 to 100,000 synapses per neuron [56]–[58]. Supplementary Figure S1 gives an alternative example where the *inhibitory* distribution is nominated as Gaussian and we solve the *excitatory* density. In both cases, the density distributions were randomly sampled to simulate the placement of excitatory and inhibitory receptors within the dendritic field (panel C). In both cases, the estimated frequency response of the combined receptor field (panel D) matched that of the target Gabor filter (panel B), as expected.

The synaptic currents were then studied whilst simulating the bombardment of the receptor field by propagating waves of cortical activity. The waves were approximated by a sinusoidal grating that propagated across the receptor field at 6 mm (20 wavelengths) per second (e.g., Figure 3E). The sinusoidal grating permitted the amplitude of the wave to be computed at exact receptor locations (e.g., Figure 3E). The amplitude of the wave modulated the rate of synaptic bombardment between 0 and 40 spikes/sec with a long-term average of 20 spikes/sec. Synaptic spikes were simulated by a Poisson process that induces an exponential rise (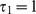 ms) and fall (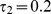 ms) in the post-synaptic conductance of the corresponding receptor (Methods, equation 10). These changes in conductance drive the synaptic currents in the membrane model (equation 1). It was found that the net synaptic current (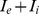) responded selectively to the orientation of the grating pattern in the receptor field, as predicted by the Gabor filter. The grating with the preferred orientation (Figure 3E) elicits significant modulation of the net balance of excitation and inhibition among the receptor conductances, resulting in large-amplitude oscillations in current (approximately 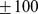 pA) as the grating propagates across the receptor field (Figure 3F). Conversely, the orthogonal grating pattern (Figure 3G) fails to modulate the conductances in any coordinated fashion hence the synaptic current remained near zero (Figure 3H). In all cases, the net inhibitory and excitatory conductances were predominantly balanced at 10 nS each, consistent with physiological observations of balanced excitation and inhibition in spontaneous cortical activity *in vivo* [59] and patch-clamped cells *in vitro* [60].

#### Two-compartment phase-only model

**Figure 4:**
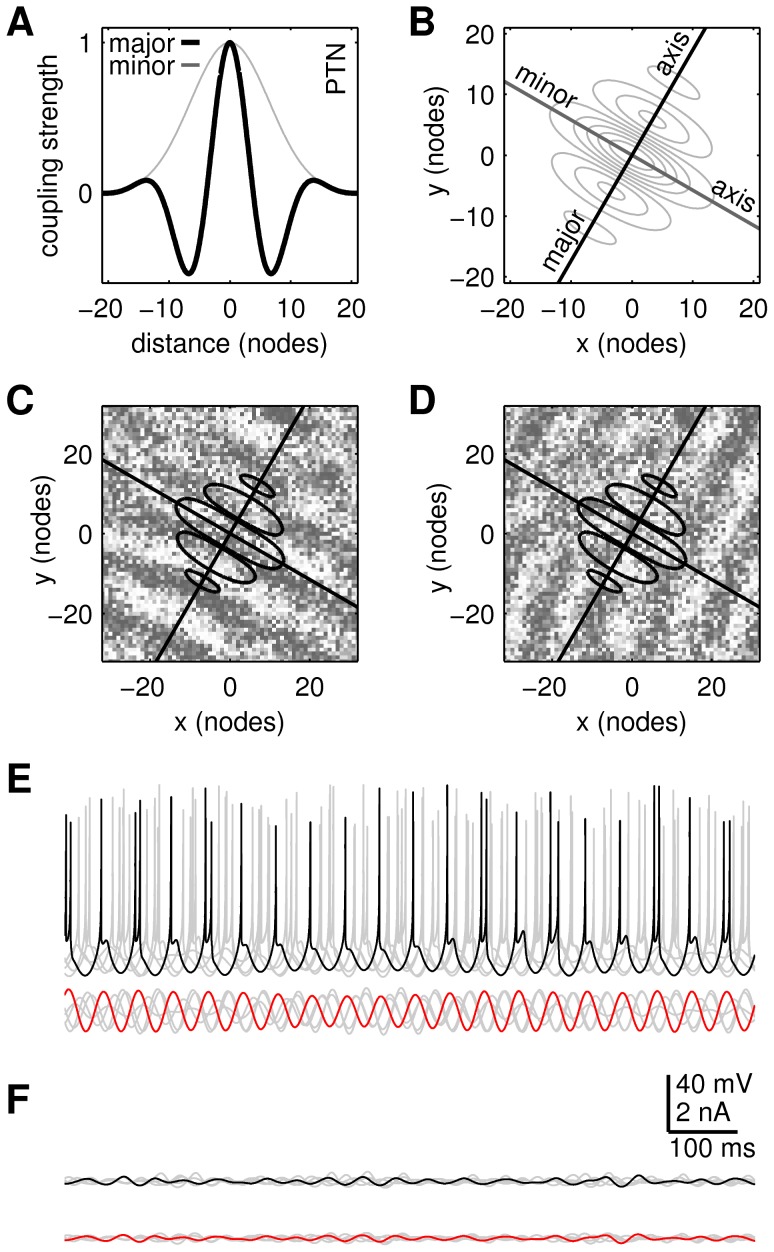
The PTN model. (**A**) Spatial profiles of the dendritic filter. (**B**) Spatial contours of the dendritic filter. (**C**) Preferred cortical oscillation pattern for this dendritic filter. (**D**) Orthogonal oscillation pattern. (**E**) Time course of the neural response to the preferred cortical pattern. Bottom trace (red) is the dendritic current. Top trace (black) is the somatic membrane potential. Light gray traces show the responses of four other PTNs located at random positions on the same cortical pattern. (**F**) Time course of neural response to the orthogonal pattern. Panels *E* and *F* have the same scales.

Having verified that dendritic receptor fields can serve as Gabor filters, we next simulated the receptor field of each PTN as a two-dimensional Gabor filter using a phase-only approach (Figure 4). Here, the output of the Gabor filter directly represents the dendritic current produced by the net synaptic bombardment of the receptor field by the local activity in the cortical oscillator model. This dendritic current flows directly into the somatic compartment where action potentials are generated according to the conductance model of Izhikevich and Edelman [61] (Methods, equations 14–15). The parameters of the somatic model were tuned to match the physiological response characteristics of pyramidal tract neurons in mammals [62]–[64].

It was found that individual PTNs responded selectively to cortical wave orientation but the response rates were limited to discrete frequency bands (10, 20, 30, 40 Hz) due to entrainment by the intrinsic 20 Hz oscillations in cortex. Typical responses of the same PTN to a pair of orthogonal wave patterns are shown in Figure 4. The response (panel E) to the preferred wave pattern (panel C) exhibits high amplitude 20 Hz oscillations in the dendritic current (red) which biases spike initiation in the somatic membrane potential (black) towards the peaks of that current. The presence of double-spikes on many of the peaks in this example resulted in a mean firing rate of 30 spikes per second. In comparison, the response (panel F) to the non-preferred wave pattern (panel D) exhibits low amplitude oscillations in the dendritic current (also at 20 Hz) which are too weak to induce any spikes in the soma.

The phase of the dendritic current is determined by the propagation of the wave pattern across the dendritic receptor field. Receptor fields placed at different cortical locations will thus respond with different temporal phase shifts. This is illustrated by the light gray spike traces in Figure 4E which show the responses of four randomly selected PTNs on the same cortical sheet.

#### Sinusoidal forcing of the soma

**Figure 5:**
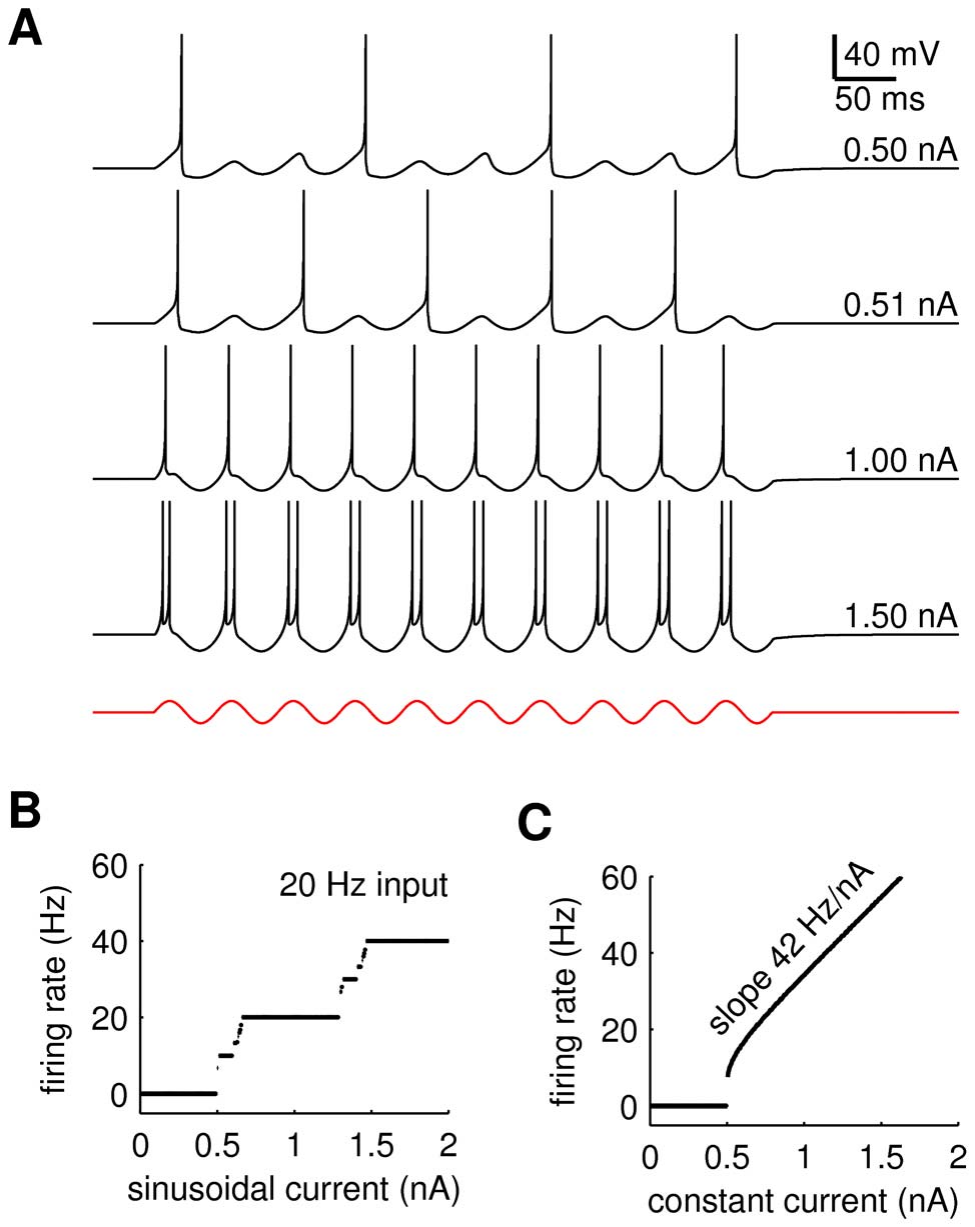
Response properties of the PTN somatic compartment. (**A**) Spike trains produced by the model in response to 20 Hz sinusoidal injection currents of amplitude 0.50 nA, 0.51 nA, 1.00 nA and 1.50 nA respectively. Bottom trace (red) shows the time course of the injection current. (**B**) Steady-state firing response of the model to 20 Hz sinusoidal injection current. The plateaus in the response curve are due to entrainment of the membrane potential to the oscillatory input. The main plateaus occur at 20 Hz and 40 Hz. Smaller plateaus also occur at 10 Hz, 13.25 Hz, 30 Hz and 33.25 Hz. (**C**) Steady-state firing response of the same model to constant injection currents. The parameters of the model were tuned so that this curve closely matched the physiological properties of pyramidal neurons [62]–[64]. Specifically, a mean slope of 42 Hz/nA and sudden onset of 10 Hz firing as the injection current approaches 0.5 nA.

The discrete response frequencies (10, 20, 30, 40 Hz) of the PTN can be understood by inspecting the response of the somatic model to a pure 20 Hz sinusoidal injection current (Figure 5A). The sinusoidal input forces an oscillation in the somatic membrane potential which is phase locked to the input. The resulting spikes tend to coincide with the rising peaks of the injection current although the number of cycles required to trigger a spike varies with current amplitude. Injection currents below 0.50 nA fail to elicit any spikes (not shown) whereas currents of 0.50 nA and 0.51 nA elicits spikes every third and second cycle respectively. At 1.00 nA the oscillating current produces regular spikes on every cycle and at higher currents (e.g. 1.50 nA) double-spikes appear.

The somatic responses to the full range of possible current amplitudes (Figure 5B) resembles the ‘devil's staircase’ of a periodically forced resonator with major frequency plateaus at 20 Hz and 40 Hz interspersed with minor plateaus at 10 Hz and 30 Hz and a multitude of even smaller m∶n phase locked solutions between those. In comparison, the somatic response to a constant injection current (Figure 5C) varies smoothly with current apart from the sudden onset of ≈10 Hz firing at 0.5 nA. This discontinuity is due to a Hopf bifurcation in the conductance-based model and it replicates the sudden onset of 10 Hz firing observed in mammalian pyramidal tract neurons [62]–[64].

#### Tuning curves

**Figure 6:**
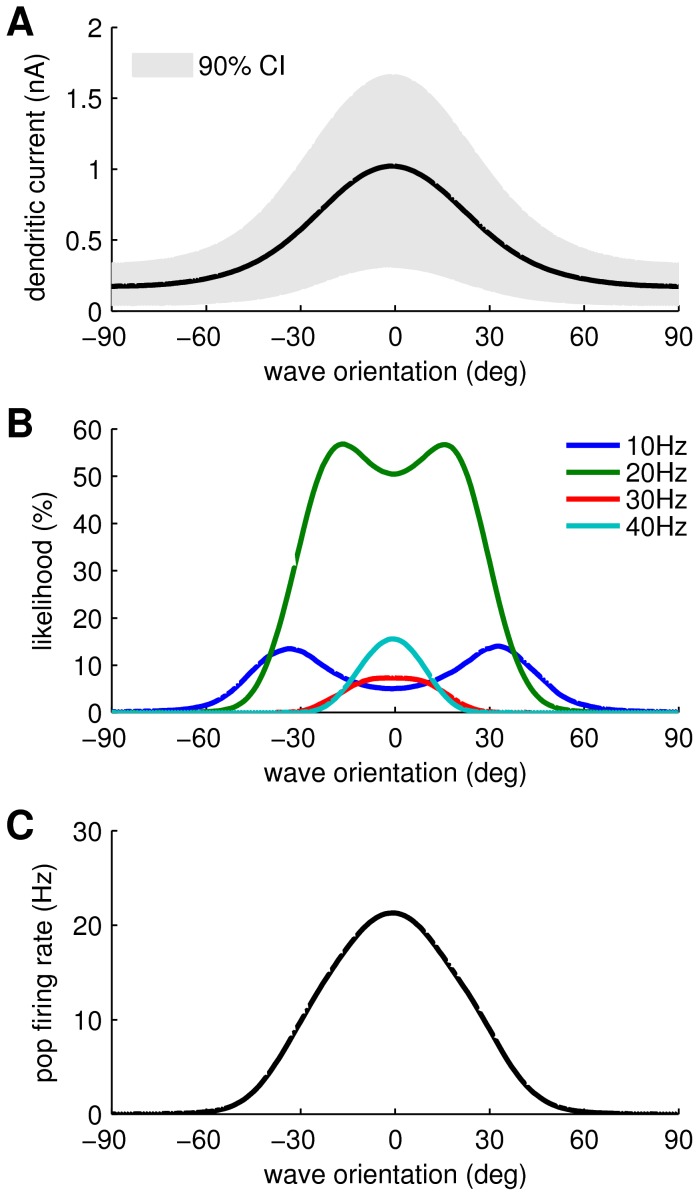
Tuning curves of the PTNs. (**A**) Tuning curve of the PTN dendritic compartment. The amplitude of the dendritic response current (vertical axis) is modulated by the orientation of the cortical wave pattern (horizontal axis). Heavy black line indicates the mean amplitude of the dendritic response for any given wave orientation. Shaded region indicates the 90% confidence interval. The large variation is due to local defects in the wave pattern. (**B**) The likelihood of the soma responding at each of the dominant firing rates. (**C**) Net firing rates of a population of neurons in response to wave orientation.

The tuning curve of the dendritic compartment (Figure 6A) was computed by averaging the dendritic responses of PTN receptor fields at all possible locations on the cortex. The measurements were repeated over n = 20 independently generated cortical wave patterns yielding a total of 327,680 samples for each wave orientation. The large variation in the dendritic responses (gray region indicates the 95% confidence interval) is due to local defects in the wave pattern.

The corresponding response frequencies of the somatic compartment (Figure 6B) were predicted by mapping the dendritic tuning curve (Figure 6A) onto the somatic response to pure 20 Hz input (Figure 5B). The result is the likelihood of the PTN firing at each of the four dominant entrainment frequencies (10 Hz, 20 Hz, 30 Hz, 40 Hz) for any given wave orientation in the cortex. Even though the dendritic tuning curve responds smoothly to wave orientation, the same is not true of the somatic compartment which responds in discrete frequency bands because of entrainment by the oscillatory input. Nonetheless a smooth tuning response is recovered by the population response of all PTNs taken together (Figure 6C). It is found that the distributions of frequency-specific responses are balanced so that the combined spike output of all PTNs is itself a smooth function of wave orientation. Notice that the population tuning curve (Figure 6C) is somewhat sharper (full width at half maximum, FWHM = 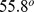) than the dendritic tuning curve (Figure 6A; FWHM = 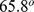). The population response is effectively zero beyond 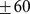 degrees thereby eliminating any response to irrelevant wave patterns.

#### Inter-spike irregularity

**Figure 7:**
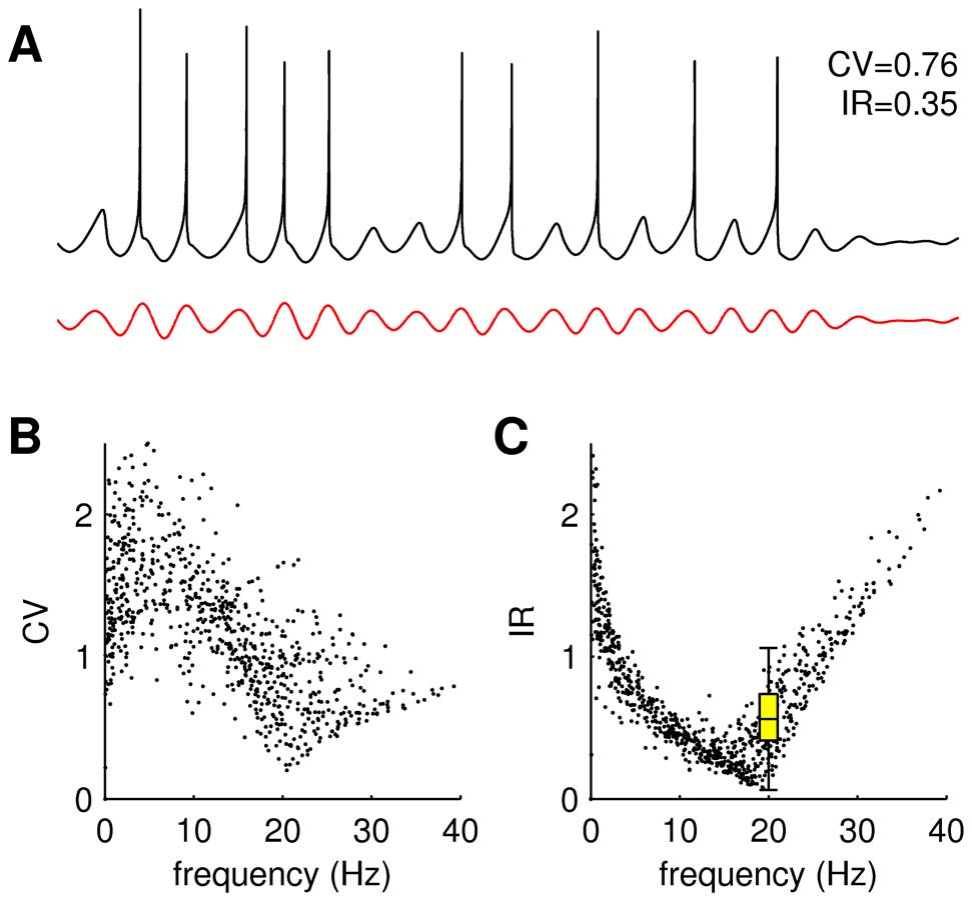
Variability of inter-spike intervals in the PTN model. (**A**) Exemplar dendritic current (red) and resulting somatic spike train (black) exhibiting irregular inter-spike intervals. The coefficient of variation (CV = 0.76) and irregularity (IR = 0.35) measures were both computed over a 30 second window. (**B**) Coefficient of variation of the inter-spike intervals versus firing rate. (**C**) Irregularity metric for the same data. Box plot (yellow) reproduces the observed irregularity of PTN inter-spike intervals in primary motor cortex [69] where the whiskers indicate the extrema.

Neurons exhibit variable inter-spike intervals *in vivo* that are difficult to replicate in purely deterministic models [65]–[67]. Inter-spike interval irregularity in the simulated PTN spike trains was quantified using both the conventional coefficient of variation (CV) and the irregularity (IR) metric (Methods, equation 20) recently proposed by Davies and colleagues [68]. Inter-spike irregularity in primate PTNs is IR≈0.6 during performance of a steady hold task [69] and similar levels of interspike irregularity were observed in our model (Figure 7). Since the PTN model contains no intrinsic source of variability, any inter-spike irregularity is entirely due to irregularity in the dendritic current (Figure 7A, red trace). That irregularity arises from the transient waxing and waning of the cortical wave pattern due to the heterogeneous oscillator frequencies in the cortical model. Transient degradations of the cortical wave pattern are reflected in weaker responses in the dendritic current. The same mechanism gives rise to the waxing and waning in the cortical LFP (Figure 2E) but in this case the oscillations are also filtered through the dendritic kernel. Interestingly, spike regularity in the model is not constant with firing rate. Inter-spike intervals become more regular (less irregular) as the spike rate approaches 20 Hz. Irregularity then returns as firing rate exceeds 20 Hz. The effect can be seen with the CV metric (Figure 7B) but is most prominent with the IR metric (Figure 7C). The minimum inter-spike irregularity at 20 Hz corresponds with 1∶1 entrainment of the soma to the oscillations in the dendritic current.

#### Phase shifts

**Figure 8:**
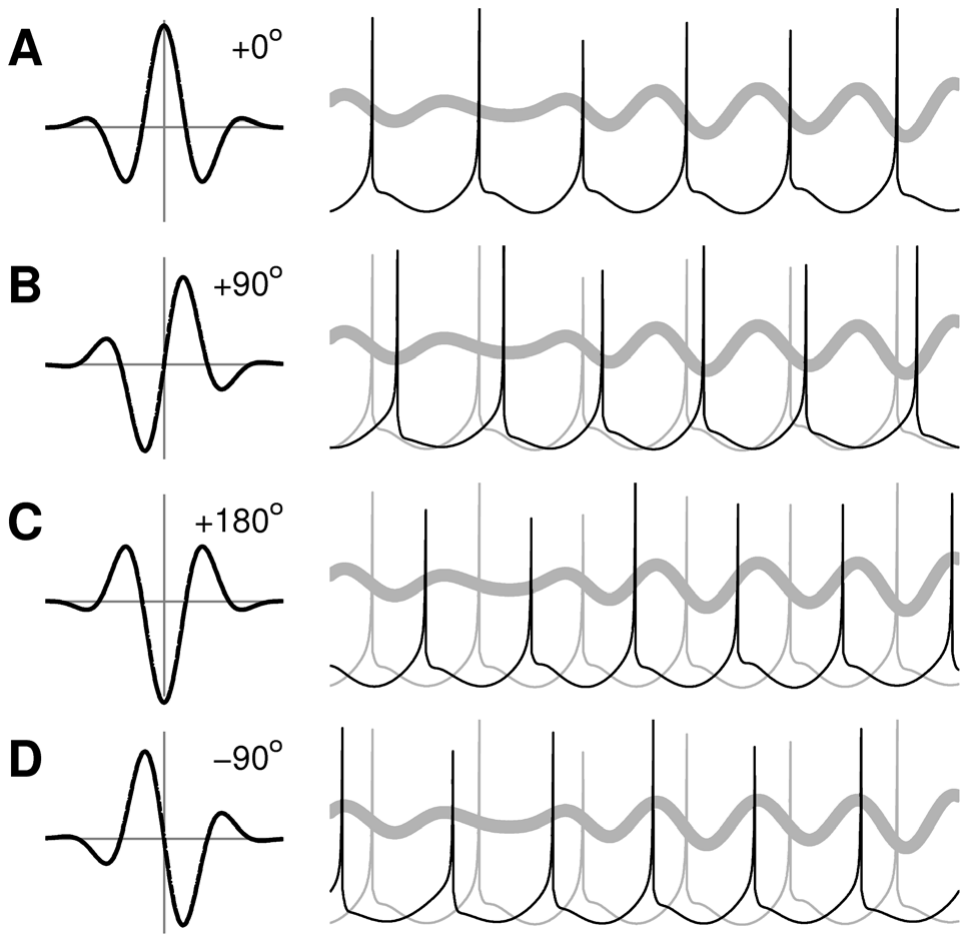
Asymmetric dendritic kernels induce phase shifts in the PTN spike trains. Profiles of the dendritic kernels are shown on the left. Spike trains produced by the PTN model are shown on the right. The thick gray line is the simulated LFP of the cortical pattern which is the same in all cases. (**A**) Case of a Gabor filter with zero phase shift. (**B**) Case of +90 degree phase shift. (**C**) Case of +180 degree phase shift. (**D**) Case of −90 degree phase shift. Light gray spike traces in B–D reproduce the case of zero phase shift for ease of comparison.

Empirical characterization of simple cells receptor cells in visual cortex [18], [19] has shown that many cells have an asymmetric profile that can be approximated by a Gabor filter with a non-zero spatial phase shift 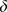 (Methods, equation 12). Signal processing theory predicts that spatial phase shifts in the Gabor filter will be transformed into temporal phase shifts in the filter output when the input is a moving sinusoidal grating, as is the case here. This prediction is confirmed in the PTN model when the spatial phase of the dendritic kernel has been shifted by 0, 90, 180 and −90 degrees respectively (Figure 8). In all cases, the spike responses of the PTN are shifted in time by the respective phase value. Neurons may thus exploit asymmetries in the spatial profile of the dendritic receptor densities in order to advance or retard spike timing relative to the incoming oscillations. This property of the dendritic filter may have relevance to coding by phase-of-firing where information is thought to be encoded in the timing of spikes relative to the phase of local oscillations [70]–[71].

### Descending motor drive

The full motor pathway from cortex to muscle (Figure 1C) was simulated to test the efficacy of translating cortical wave patterns into muscle activity. A pool of identical PTNs (n = 200) were placed randomly in the motor cortex. Following the methods of [41], the outputs of the PTNs were randomly connected to a pool of MNs (m = 100) such that each MN received input from exactly 60 PTNs with the likelihood that any two MNs had 30% of their inputs in common. MNs were modeled as leaky integrate-and-fire neurons with stochastic membrane thresholds (Methods, equations 16–17). The output spikes were convolved with heterogeneous motor unit action potentials (MUAP) to simulate the surface electromyogram (EMG) of muscle (Methods, equations 18–19). Muscle force was not modeled directly but was inferred from the amplitude envelope of the simulated EMG.

**Figure 9:**
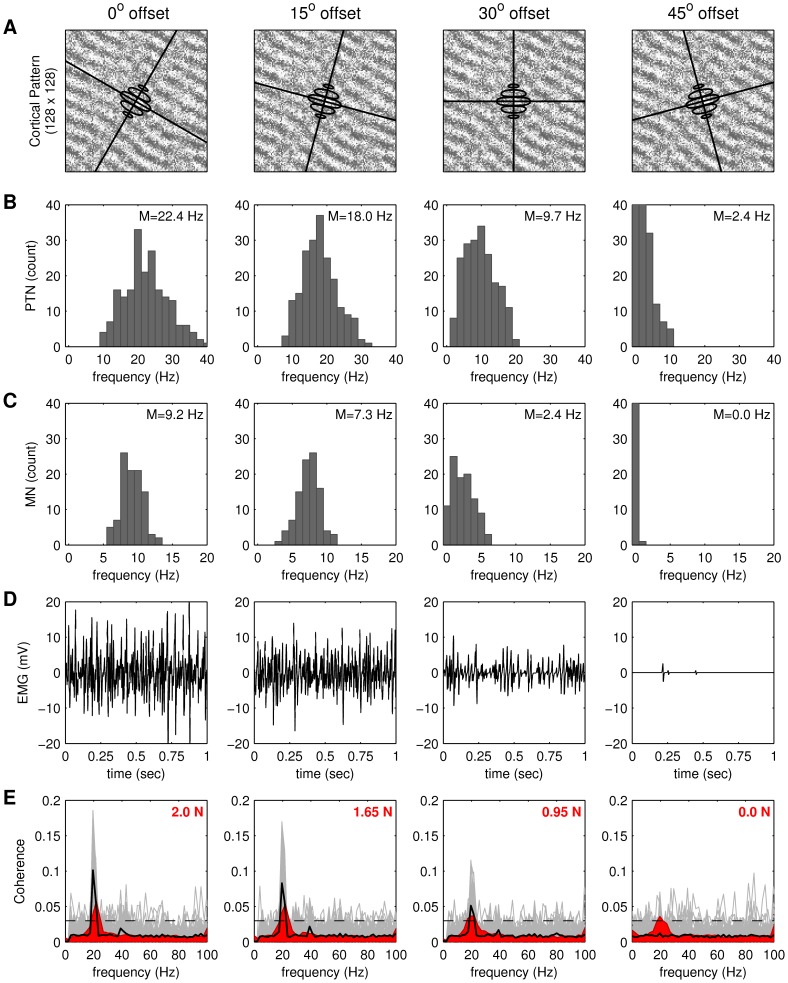
The effect of wave orientation on the output of the descending motor system. Each column presents the responses of the descending motor system for pyramidal neurons with a given dendritic orientation (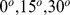 and 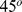) relative to the cortical pattern. (**A**) Orientation of the dendritic kernels. The cortical pattern is the same in all cases. (**B**) Firing rate distribution of the pyramidal tract neurons. (**C**) Firing rate distribution of the motor neurons. (**D**) Time course of the simulated EMG. (**E**) Magnitude squared coherence between LFP and EMG. Light gray lines represent individual trials (n = 100). Black line shows the trial average. In red, average MEG-EMG coherence in 16 subjects while they perform a precision grip task at different force levels (2.0 N, 1.65 N, 0.95 N, 0.0 N). Dashed horizontal line indicates the 95% confidence level for the coherence distribution in each frequency bin. Peaks above that line are statistically significant at *p = 0.05*.

Thirty seconds of cortical traveling wave activity was simulated using a fixed cortical coupling kernel that was oriented at 60 degrees from the horizontal (as in Figure 2). This wave sequence was then decoded by PTNs with dendritic filters that were rotated away from the dominant wave orientation by 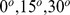 and 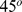 respectively in each condition. The results are rotationally equivalent to holding the orientation of the dendritic filters fixed while manipulating the orientation of the cortical coupling except in this case there are no confounds with between-trial differences in the self-organized wave patterns. Figure 9 shows various aspects of the descending motor drive for each orientation offset condition. Each column pertains to one condition. The panels in row *A* show the orientation of each of the dendritic filters in relation to the cortical wave pattern. The panels in row *B* show the distribution of firing rates exhibited by the 200 PTNs embedded in the cortex. The mean firing rates of the PTN population (22.4 Hz, 18.0 Hz, 9.7 Hz, 2.4 Hz) are seen to diminish as orientation offset increases from 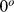 to 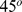 which confirms that PTN responses are selective to wave orientation. The maximum responses occur when the waves are perfectly aligned with the dendritic filter (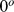 offset) whereas the bulk of the PTNs barely fire at all in the case of 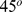 offset. A persistent spread in the PTN response rates is observed for all orientation offsets. This variation is due to local defects in the wave pattern, as will be discussed later.

Figure 9C shows the distribution of firing rates for the spinal motor neurons (MN). They too exhibit diminished responses as the orientation offset of the dendritic filter is increased. Here the mean firing rates of the motor neuron pool diminishes from 9.2 Hz at zero offset down to virtually no response at 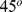 offset. The absence of all but a few random spikes in the motor output in the latter case show that the background PTN spikes are insufficient to raise the motor neuron membrane potential above its firing threshold. The simulated EMG traces (Figure 9D) represent the net motor neuron activity as it would be observed at the surface of the muscle. Once again, the response is diminished with increased offset angle between the cortical waves and the dendritic filter. The EMG amplitude may be loosely interpreted as indicative of muscle contractile force.

Lastly, the average coherence between LFP and EMG over 100 randomized trials (Figure 9E; heavy black line) reveals weak but significant 20 Hz corticospinal coherence that also diminishes with offset angle. This reduction of simulated coherence with offset angle is consistent with reduced corticospinal coherence in human MEG/EEG under reduced levels of muscle force (Figure 9E; red). The weakness in the levels of simulated coherence are also consistent with physiological reports of coherence in the range 0.01–0.1 [39], [44], [72]. In our model, this weakness is a direct result of the heterogeneity among the cortical oscillator frequencies (Figure 2D). Eliminating that heterogeneity leads to stronger coherence values (approaching 0.5).

## Discussion

We present a novel solution to the problem of encoding and decoding motor commands in primary motor cortex using spatiotemporal patterns of beta oscillations. In particular, we propose that motor commands encoded in the morphology of traveling waves can be discriminated (decoded) by the dendritic arbors of PTNs to selectively engage spinal motor neurons, thereby orchestrating muscle movement. Our model demonstrates a unique mechanism by which spatiotemporal patterns in cortex exert control over muscle activity while also replicating key aspects of the descending motor system, including variable inter-spike intervals and weak corticospinal coherence during steady motor tasks.

The key aspect of the proposed model is the formulation of the dendritic receptor field as a filter of spatial patterns in the phase of incoming oscillatory signals. In this view, neural information is encoded by the relative timing of the synaptic input. Dendritic computation is thus portrayed as the integration of synaptic phases rather than the integration of synaptic membrane potentials. That is not to say the model confounds phase with membrane potential but rather that it emphasizes the *impact* of the synaptic phase on the timing of subsequent spikes produced by the neuron. This phase-based approach is consistent with emerging evidence that dendritic integration is sensitive to the relative timing and spatial location of synaptic input on the dendritic arbors [21], [22], [27], [28], [31]–[33]. Phase-only models are justified in studies of neuronal synchronization where the timing of synaptic input is of prime importance [46]–[50], [73]–[76].

We approximated the spatial integration of synaptic input across the dendritic receptor field using a two-dimensional Gabor filter. The spatial bandpass characteristics of Gabor filters are well understood and have previously been used to characterize receptor fields in visual cortex [18], [19]. In those cases, the Gabor filtering refers to the spatial properties the stimulus rather than the spatial properties of the activity patterns in the visual cortex. Nonetheless, the retinotopic map of the visual field is locally preserved in visual cortex (e.g., [77]) so it is reasonable to consider that Gabor filtering may apply at the level of cortical activity patterns. We assume that similar structures may plausibly occur in motor cortex although we are not aware of any direct experimental reports of such. Furthermore, our simulations show that the spatial arrangement of excitatory and inhibitory receptors within the dendritic field is sufficient for the neuron to act as a Gabor filter of spatial patterns of synaptic bombardment. Whilst we suggest that such excitatory and inhibitory inputs arise from local interneurons, it is also possible that such effects reflect restricted corticothalamic circuits, which are known to contribute to the response properties of visual cortical neurons [78]–[80]. We do not propose a specific explanation of how such spatially organized receptor fields may develop, except to recall that a homeostatic balance between excitation and inhibition does appear to be actively maintained by a regulatory push-pull mechanism at the level of the dendrite [60]. We also note that traveling waves have themselves been implicated in guiding the development of neuronal circuits in cerebellar cortex [81]. In such cases, spontaneously generated internal activity is thought to serve as a means of bootstrapping the development of cortical circuitry prior to the onset of sensory experience [82].

Some studies of dendritic morphology in visual cortex have previously dismissed any relationship between the morphology of the dendritic footprint and the functional selectivity of those cells to the orientation [83] or direction [84] of visual stimuli. However, those studies [83], [84] only considered the physical shape of the dendritic field and not the spatial densities of the receptors within it. We emphasize that it is the spatial distribution of excitatory and inhibitory synapses that is key to our findings, not the physical shape of the dendritic footprint. Our findings also show that asymmetrical placement of the dendritic receptors can shift the temporal phase response of cells by up to 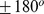 even though the underlying footprint of the receptor field is unchanged. We suggest this mechanism may be exploited by the brain to fine tune the timing of spikes relative the phase of local oscillations for such purposes as long-range neural coordination [85] or coding by phase-of-firing [70], [71]. We anticipate that the same underlying mechanism would apply to any spatial filter which has periodic modulation on finite support, not just Gabor filters. Ultimately, the veracity of any computational study rests upon the validity of its core assumptions as well as the degree to which such assumptions can be verified or refuted by independent measurement. Here the core assumption is that the spatial distribution of excitatory and inhibitory synapses across a dendritic tree serve as a spatial filter, transforming spatiotemporal patterns of local oscillatory activity in motor cortex into oscillatory changes in the soma potential and thence into periodically-modulated spike sequences in Betz cells. This lends itself to several lines of independent inquiry, including in vivo measurements that couple multi-channel measurements of local field potential to spike activity, as well as morphological characterization. Computational studies such as the present one may hence guide empirical research by providing quantitative predictions that allow differentiating between alternative competing computational frameworks. In the absence of such an approach, the level of detailed description regarding dendritic computation will remain confined to the microscopic scale, leaving macroscopic accounts reliant upon qualitative heuristics.

Although the ability of dendrites to discriminate specific temporal sequences of synaptic inputs has previously been investigated [28], [31], [33] there is relatively little research exploring the potential of dendrites to discriminate spatial patterns of oscillatory inputs. Oscillatory neural signals are key to many cognitive and behavioral processes [86]–[89] and beta oscillations in motor cortex have long been implicated in movement [44], [45], [72]. The spatial organization of those oscillations as traveling waves is only a recent discovery [10]–[12] and the present model demonstrates a plausible neural architecture for transforming small-scale (sub-millimeter) spatiotemporal activity patterns into steady muscle activity. However the present model does not account for decoding the large-scale wavelengths (1 cm) observed in motor cortex since such wavelengths far exceed the spatial resolution of individual PTN receptor fields.

In our model, oscillatory activity in cortex is translated into steady motor output. The long-term motor output remains constant for any given wave pattern, exhibiting only random fluctuations about the mean due to stochastic influences within the motor pool. Despite this overall constancy, echoes of the cortical oscillations are still transmitted through the descending motor pathways where they are observed in the model as weak levels of 20 Hz coherence between the LFP and the EMG. These simulated findings are consistent with the weak but significant levels of corticospinal coherence observed in humans and primates during steady motor tasks [42]–[44]. Motor neurons transmit oscillatory activity to the muscle almost linearly hence the weakness of the neurobiological levels of coherence must be due to degradation of the oscillatory signals in the corticospinal drive[41], [90]. In many computational models, that degradation is replicated using injected noise. In the present model it arises deterministically from the heterogeneity of the cortical oscillators without resort to explicit stochastic terms.

Oscillator heterogeneity plays an important role in the present model. Firstly it demonstrates that pattern formation and discrimination remains feasible even when the intrinsic oscillation frequencies are broadly distributed, as in the beta bandwidth (12–20 Hz). Secondly, it injects significant ongoing variability into the cortical patterns which becomes evident in the irregular PTN inter-spike intervals and the waxing and waning of the simulated LFP. The gradual reduction in simulated inter-spike variability as PTN firing rates increase from 0 Hz to 20 Hz is broadly consistent with observations in motor neurons where variability typically decreases from CV≈0.4 near 7 Hz firing to CV≈0.2 near 20 Hz firing [91], [92]. In the model, that variability arises deterministically from transient synchronizations among the cortical oscillators. In dynamical systems theory, this phenomenon is referred to as *metastability* because the transient states are not strictly stable but the dwell-time in the vicinity of these states is sufficiently long that they appear stable in the short-term. The richness of brain dynamics is often attributed to metastability [93]–[95] although demonstrations of metastability in neural models with an explicit functional role, such as the present, are rare.

Oscillations in our cortical model also has the effect of entraining the output spikes of individual PTNs into discrete frequency bands. This leads to step-wise increments in PTN firing frequency that would appear to counteract the ability of the PTNs to respond smoothly to changes in the cortical patterns. Nevertheless, a smooth tuning curve is recovered at the population level where the collective responses of all PTNs yields a smooth tuning curve that actually has a sharper cut-off than the constituent dendritic filters. This type of population-level response is consistent with the population code hypothesis proposed by Georgopolous and colleagues whereby specific movements are not encoded by individual neurons in motor cortex but in the collective responses of multiple neurons each with overlapping tuning curves [96].

The simulated waves in the present model only serve as a gross approximation of the traveling waves observed in motor cortex [10]–[12]. While spontaneous and stimulus-evoked waves are both observed during the planning and execution stages of movement, only the phase and amplitude of the stimulus-evoked waves have been successfully correlated with movement. It is possible that movement may also correlate with other wave features that are not are not time-locked to behavioral cues and so are not detected by these experimental techniques [10]. Nonetheless, the present interpretation of wave orientation as encoding specific motor commands is deliberately simplified. Waves in motor cortex can propagate in any direction but predominantly propagate along the rostral-caudal axis in monkeys [10], [11] and the medial-lateral axis in humans [12]. Moreover these traveling waves tend to be solitary waves — perhaps better called wave fronts — rather than the tiled wave patterns presented here. In humans, the medial-lateral propagation direction corresponds with the somatopic progression of the motor map. Consequently, it has been suggested that wave fronts may coordinate the proximal-to-distal sequencing of muscle recruitment that is common to many types of limb movement [97]. Reconciling our model with these recent empirical observations and their heuristic interpretation [97] would suggest that the very long wave front along motor cortex [10]–[12] heralds a sweep through a sequence of movements, whereas each specific movement command nested within this sequence is encoded according to local patches of continuously propagating wavefronts. Such a hierarchy of movement sequences is consistent with other accounts of complex behavior control [98] and indeed general principles of cortical dynamics [99].

Traveling waves are not restricted to motor cortex and the proposed dendritic mechanism may also generalize to traveling waves in other modalities, such as olfactory cortex [3] or visual cortex [7]. At a deeper level, traveling waves are just one specific example of spatially embedded ensemble activity. Greater information capacity could be achieved using more complex spatiotemporal patterns of activity, hence speaking to a broader computational principle, consistent with recent work showing that the spiking behavior can be predicted from its surrounding local field potential [100], [101].

In conclusion, we propose an integrated and novel account for both encoding and decoding motor commands in motor cortex, incorporating basic histological and neurophysiological data into our model. Whilst somewhat speculative — by necessity — our model makes specific predictions regarding the organization of neuronal activity during movement and the fine-grained histology of PTNs, which lend themselves to empirical testing. There exist few other computational accounts of dendritic filtering that explicitly accommodate the oscillatory nature of spatiotemporal neuronal activity. We concede that the exact encoding of motor commands likely diverges somewhat from our present abstraction. Nonetheless, we anticipate that dendritic trees are capable of filtering a broader class of oscillatory spatiotemporal patterns than those we have investigated here. By proposing a formal account that links the information available in spatially organized oscillatory activity to the architecture of dendritic arborization, we suggest a deeper computational principle that may apply more generally in the cortex.

## Methods

### Cortical model

Motor cortex was modeled as a two-dimensional array of spatially coupled Kuramoto [54] oscillators
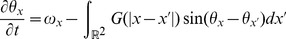
(2)with periodic boundary conditions. The phase 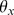 of each oscillator represents the oscillatory neural activity of a small patch of motor cortex at spatial position 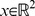. The frequency 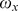 of each oscillator was drawn randomly from a normal distribution (M = 20 Hz, SD = 4 Hz) that approximates the beta bandwidth of oscillation frequencies. Center-surround spatial coupling was approximated by an anisotropic kernel,
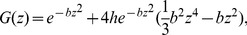
(3)based on the fourth derivative of a Gaussian surface where 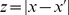 represents spatial distance. The kernel parameter 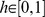 dictates the strength of the inhibitory surround as shown in Figures 2A and 2B. The inhibitory strength varies radially according to
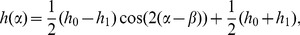
(4)where 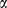 is the angular position of each oscillator relative to the kernel midpoint and 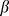 is the orientation of the major axis of the kernel itself. Parameters 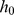 and 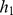 thus define the inhibitory strengths along the major and minor axes of the kernel respectively. The kernel is isometric when 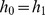. We have previously reported waves and uniform synchrony for this type of spatial coupling with parameter values in the range 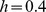 to 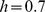 [17].

In the present study, the strength of the inhibitory surround was fixed at 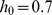 and 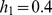 to produce traveling waves that were spatially aligned with the kernel axes (e.g. Figure 2C). The size of the coupling kernel was fixed at 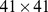 nodes with a Gaussian full-width-half-height of 11 nodes (i.e. 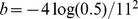). See [17] for details of the numerical integration method.

### Local Field Potential (LFP)

The LFP of motor cortex was approximated by treating the cosine of the oscillator phases as analogous to membrane voltage potential and then summing those voltages across space,
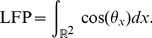
(5)Spectral density estimates of the LFP were computed using Welch's periodogram method with a Hamming window of 0.5 seconds and 50% window overlap. Sampling frequency was 1000 Hz.

### Pyramidal Tract Neuron (PTN)

Pyramidal tract neurons were modeled as two passively coupled neural compartments. The first compartment represents the dendritic tree which defines the spatial distribution of incoming connections from the motor cortex. The second compartment represents the soma which defines the spiking output of the neuron in response to the dendritic current. The dendritic current was simulated in two ways. The first method demonstrates the principle of Gabor filtering by dendritic receptors. This is achieved by a conductance-based model of the post-synaptic currents produced by spatially distributed populations of excitatory and inhibitory synapses. The second method applies those findings to the simulation of multiple PTNs in a computationally efficient manner. This is achieved by a phase-only model of the dendritic compartment in which a Gabor filter directly transforms incoming wave patterns into an oscillatory dendritic current.

#### Dendritic conductance model

The net current flowing into the dendritic compartment was modeled by the membrane equation,
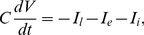
(6)where 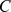 is the membrane capacitance, 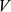 is the membrane potential, 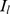 is the membrane leak current, 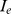 and 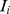 are the net currents of the excitatory and inhibitory synapse populations respectively. The leak current,
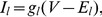
(7)was modeled with a fixed conductance 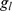 with a reversal potential of 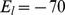 mV. The excitatory and inhibitory synaptic currents,
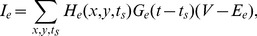
(8)

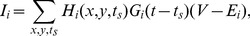
(9)were modeled with time-dependent post-synaptic conductances, 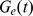 and 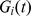, with synaptic reversal potentials of 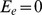 mV and 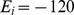 mV respectively. The Heaviside function, 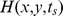, represents synaptic spike events at spatial position 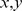 which are onset at time 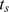. Synaptic bombardment of the dendrite was simulated by a Poisson process that was rate-modulated between 0 and 40 Hz according to the amplitude of a sinusoidal grating of wavelength 300 µm that propagated at 6 mm/sec. The sinusoidal grating represented background traveling wave activity which oscillated at 20 Hz on average. Each synaptic spike (Poisson event) produced an exponential rise-and-fall in the post-synaptic conductance governed by
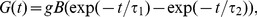
(10)where 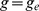 and 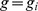 are the peak conductances of the excitatory and inhibitory receptors and 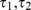 are exponential rise and fall times. The time courses of the excitatory and inhibitory synapses were identical. In both cases the peak synaptic conductance occurs at at 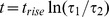 where
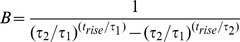
(11)and 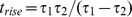 are scaling constants [102]. See Table 1 for the full list of parameter values for the conductance model.

**Table 1:** Parameters of the dendritic conductance model.

Parameter	Description
*C* = 80	Membrane capacitance (pF)
*g_l_* = 0.01	Net leak conductance (nS)
*g_e_* = 0.01	Conductance of each excitatory receptor (nS)
*g_i_* = 0.01	Conductance of each inhibitory receptor (nS)
*E_l_* = −70	Reversal potential of the leak (mV)
*E_e_* = 0	Reversal potential of the excitatory receptors (mV)
*E_i_* = −120	Reversal potential of the inhibitory receptors (mV)
*τ*_1_ = 1.0	Rise time of the synaptic conductance (ms)
*τ*_2_ = 0.2	Fall time of the synaptic conductance (ms)
*dt* = 0.1	Integration time step (msec)

The spatial distributions of the excitatory and inhibitory synapses (Figure 3A) were chosen to combine as the two-dimensional Gabor filter,
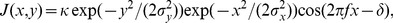
(12)where 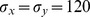 µm dictates the width of the Gaussian envelope and 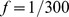 cycles/µm is the frequency of the spatial periodicity. The phase shift of the spatial periodicity was fixed at 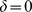. These parameter values satisfy reasonable biological limits and also match the the shape of Gabor filters observed in the simple cells of visual cortex [19]. The peak density of the Gabor function was scaled to 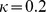 synapses per 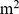. Doing so fixed the ratios of the peak densities of the constituent inhibitory and excitatory receptor distributions to within biologically plausible ranges.

A wide range of excitatory and inhibitory density distributions can be combined to satisfy the target Gabor filter (equation 12). Specific solutions were obtained by nominating a Gaussian distribution for one of the receptor populations and then solving the density distribution of the other receptor population. That was achieved in the Fourier domain by subtracting the frequency response of the known receptor distribution from the frequency response of the target Gabor filter. The inverse Fourier transform converts that solution back to the spatial domain. In Figure 3A, the distribution of the excitatory receptors was constrained to a symmetric Gaussian distribution (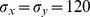) with a peak density of 0.4 synapses/µm^2^. In supplementary Figure S1, it was the inhibitory distribution that was constrained to a Gaussian (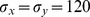) with a peak density of 0.3 synapses/µm^2^.

Each of the density distributions for the excitatory and inhibitory receptor populations were then randomly sampled to generate a set of receptor locations (x,y) within the dendritic field (e.g., Figure 3C). The number of samples drawn from each distribution was, by definition, equal to the volume circumscribed by the density distribution. In all cases, the total number of synapses in the receptor field fell within physiological estimates of 60,000 to 100,000 synapses per neuron [56]–[58]. The frequency response of the combined receptor field (x,y) was computed by convolving the excitatory receptor responses (+1) and the inhibitory receptor responses (−1) with the spatial grating over a range of grating frequencies 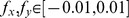 cycles/µm (e.g., Figure 3D).

#### Dendritic compartment phase-only model

In the phase-only approximation of the dendritic compartment, the net synaptic current flowing into the dendrite was conceptualized a weighted sum of the cosine of the oscillator phases,
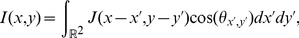
(13)where 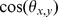 approximates the oscillatory pre-synaptic input at receptor location (x,y) and 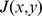 is the Gabor formulation (equation 12) of the receptor field. All PTNs were assumed to have identical receptor fields (Figures 4A and 4B) apart from rotation in the x,y plane. The spatial frequency 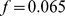 (cycles/node) of the Gabor filter was chosen to match the dominant spatial frequency of the traveling waves produced by the cortical model. The width of the Gaussian envelope was fixed at 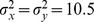 nodes. The Gabor scaling parameter 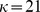 was chosen so that the receptor field responds to its preferred cortical pattern with a maximal dendritic current of 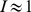 nA. The phase shift 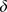 was only used to construct the asymmetric Gabor filters shown in Figure 8. In all other cases it was fixed at 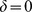.

#### Somatic compartment

The somatic compartment of the PTN model was implemented using the conductance model of Izhikevich and Edelman [61],
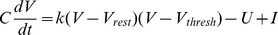
(14)

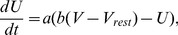
(15)where 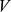 is membrane potential (mV), 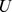 is the recovery current (pA) and 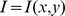 is the dendritic current (pA). The soma is considered to have spiked whenever 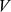 exceeds 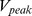. The reset conditions 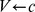 and 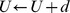 are then applied. The parameters of the Izhikevich and Edelman model [61] are described in Table 2. The values of these parameters were tuned so that the firing response to constant injection current (Figure 5C) closely matched that of pyramidal cells observed *in vivo* [62] and *in vitro* [63], [64].

**Table 2:** Parameters of the PTN soma model.

Parameter	Description
*C* = 80	Membrane capacitance (pF)
*k* = 4	Dimensionless parameter
*V_rest_* = −70	Membrane resting potential (mV)
*V_thresh_* = −50	Membrane threshold potential (mV)
*V_peak_* = 50	Membrane spike peak (mV)
*a* = 0.04	Dimensionless parameter
*b* = 10	Dimensionless parameter
*c* = −60	Membrane reset potential (mV)
*d* = 800	Recovery reset parameter (pA)
*dt* = 0.1	Integration time step (msec)

### Motor neuron (MN)

To allow comparisons between our model of the descending motor system and known physiological properties of spinal motor neurons, we simulated a motor neuron pool that received incoming spikes from n = 200 PTNs that were randomly distributed on the model cortex. The motor neuron pool was modeled as n = 100 leaky integrate-and-fire neurons with stochastic membrane resets using the same method as [41]. The membrane potential 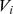 of each MN was thus modeled as,
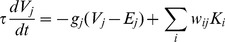
(16)where 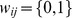 is an all-or-none connection from Betz cell 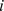 to motor neuron 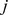 and 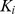 is the post-synaptic current generated by the incoming spikes. The latter has exponential rise and fall,
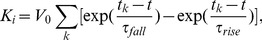
(17)where 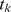 denotes the time of the 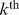 spike and the 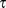 terms are time constants. All other parameters are described in Table 3.

**Table 3:** Parameters of the MN model.

Parameter	Description
*V_j_*	Membrane potential (mV)
*E_j_* = −70±1	Equilibrium potential (mV)
*g_j_* = 1±0.167	Leakage conductance (pS)
*w_ij_*	Connection weight (0 or 1)
*K_i_*	Post-synaptic potential
*V*_0_ = 20	Post-synaptic scaling constant
*τ* = 10±3.33	Membrane time constant (ms)
*τ_rise_* = 1	Post-synaptic rise time (ms)
*τ_fall_* = 3	Post-synaptic fall time (ms)

Parameter values marked with ± are drawn from a normal distribution where the error term indicates standard deviation.

The connection weights 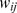 were arranged so that each MN received input from exactly 60 PTNs. These were randomly assigned with the proviso that any two MNs would, on average, share 30% of their inputs with a common set of PTNs [41], [90].

### Electromyograph (EMG)

The simulated EMG produced by the motor unit pool was obtained by convolving the motor neuron output spikes with a biologically realistic motor unit action potential (MUAP) and summing the result across all motor neurons. The MUAP was defined as
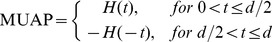
(18)where
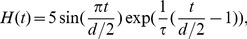
(19)is a conventional bi-phasic pulse with a time constant of 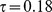 ms and duration of 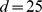 ms [90]. The amplitude of the MUAP for each motor neuron was randomly scaled between 0 and 1 to reflect natural variation in size and location of muscle fibers. Likewise, the polarity of the MUAP was inverted for randomly selected motor neurons. See [41] for the benefits of modeling heterogeneous motor action potentials.

### Corticospinal coherence

Corticospinal coherence measures the degree by which oscillations in the EMG can be predicted by those in the LFP. It has become an important tool in exploring corticospinal interactions in motor control (see [72]). Weak but significant levels of coherence between 0.01 and 0.1 are typically observed in the beta bandwidth during steady hold tasks (e.g. [44]). We approximated corticospinal coherence by the magnitude squared coherence of the simulated LFP and EMG signals over a 30 sec data window. The coherence spectra were computed using Welch's periodogram method with a Hamming window of 0.5 sec and 50% window overlap. The 95% confidence level for the resulting coherence spectrum is 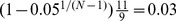 where *N* = 120 is the total number of data windows [103], [104]. Both the EMG and coherence spectra were averaged over 100 repeat simulations to control for variation in the model parameters and stochasticity in the motor neuron model.

### Irregularity metric (IR)

The variability of inter-spike intervals was quantified using both the conventional coefficient of variation (CV) metric and the irregularity (IR) metric,
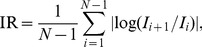
(20)proposed by Davies and colleagues [68]. The latter emphasizes relative changes in consecutive inter-spike intervals (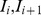) and is more resistant to changes in firing rate than the coefficient of variation. We sought similar levels of inter-spike irregularity (IR≈0.6) to those reported in the PTNs of monkey primary motor cortex during a precision hold task [69].

## Supporting Information

Figure S1 **An alternative example of Gabor filtering by dendritic receptor densities.** All panels are the same as in Figure 3 except in this case the inhibitory, rather than the excitatory, receptor density distribution was nominated as Gaussian (panel A). Consequently the receptor fields (panel C) differ from that of Figure 3 but the frequency responses (panels B and D) do not. Once again, the dendritic current is modulated by the preferred wave pattern (panels E–F) but not the orthogonal wave pattern (panels G–H). This alternative combination of receptor densities provides another example of Gabor filtering.
(TIFF)
