# Supplementary figures and images for: A Dendritic Mechanism for Decoding Traveling Waves: Principles and Applications to Motor Cortex
Source: PLoS Comput Biol. 2013 Oct 31;9(10):e1003260. doi: 10.1371/journal.pcbi.1003260 (PMC3814333; doi:10.1371/journal.pcbi.1003260)

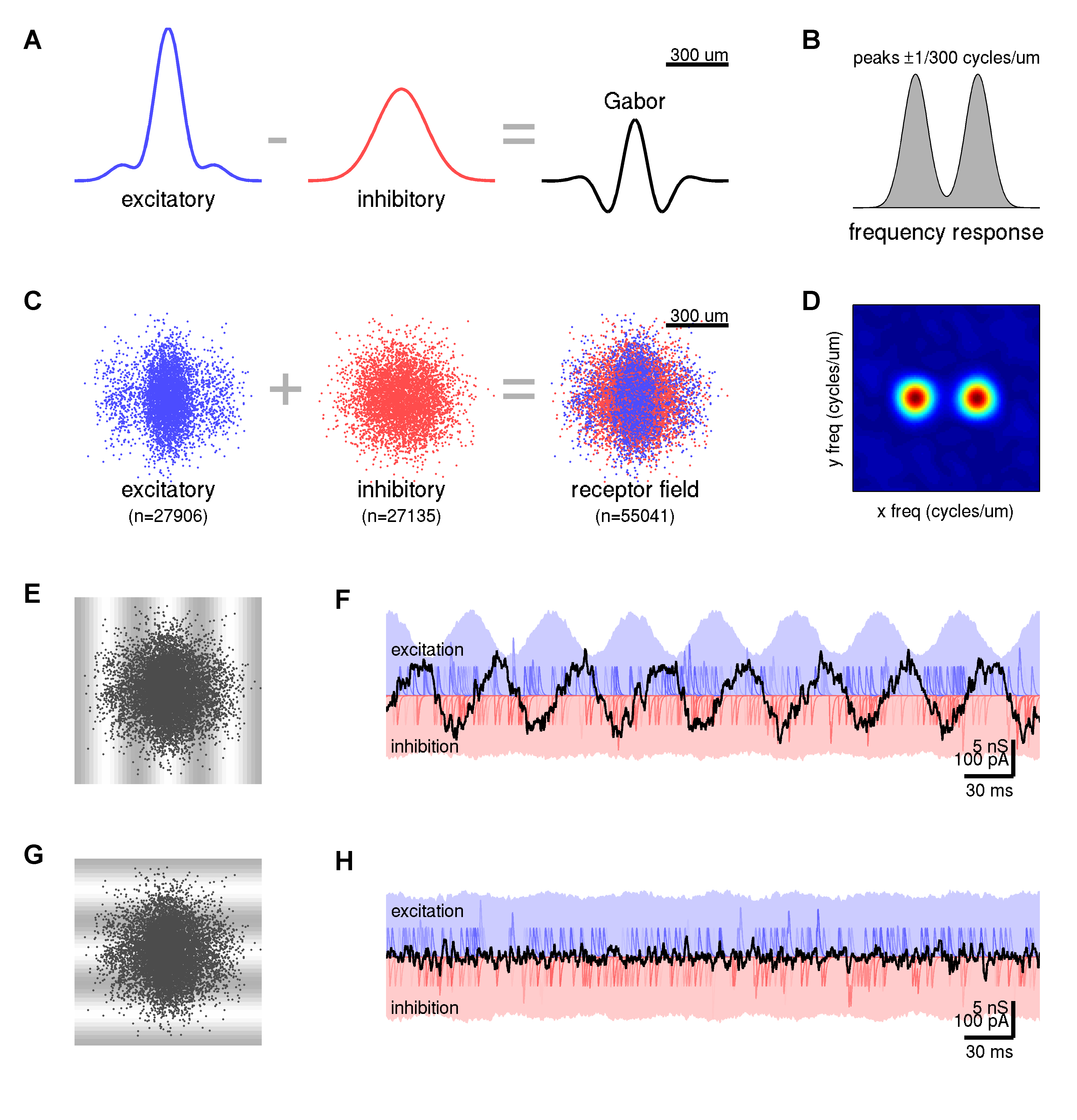

Supplement: Figure S1 — An alternative example of Gabor filtering by dendritic receptor densities. All panels are the same as in Figure 3 except in this case the inhibitory, rather than the excitatory, receptor density distribution was nominated as Gaussian (panel A). Consequently the receptor fields (panel C) differ from that of Figure 3 but the frequency responses (panels B and D) do not. Once again, the dendritic current is modulated by the preferred wave pattern (panels E–F) but not the orthogonal wave pattern (panels G–H). This alternative combination of receptor densities provides another example of Gabor filtering. (TIFF) [file pcbi.1003260.s001.tiff]
